# Clinical features and management of medication-induced persistent corneal epithelial defects due to topical ophthalmic medications

**DOI:** 10.3389/fmed.2026.1928830

**Published:** 2026-09-25

**Authors:** Shunrong Luo, Nuozhou Wu, Yuan Lin, Huping Wu

**Affiliations:** 1 Xiamen Eye Center and Eye Institute of Xiamen University, School of Medicine, Xiamen, China; 2 Xiamen Clinical Research Center for Eye Diseases, Xiamen, Fujian, China; 3 Xiamen Key Laboratory of Ophthalmology, Xiamen, Fujian, China; 4 Fujian Key Laboratory of Corneal & Ocular Surface Diseases, Xiamen, Fujian, China; 5 Xiamen Key Laboratory of Corneal & Ocular Surface Diseases, Xiamen, Fujian, China; 6 Translational Medicine Institute of Xiamen Eye Center of Xiamen University, Xiamen, Fujian, China; 7 Department of Ophthalmology, The First Affiliated Hospital, Fujian Medical University, Fuzhou, Fujian, China

**Keywords:** adverse ophthalmic medication, corneal wound healing, *in vivo* confocal microscopy, medication-induced persistent corneal epithelial defect, ocular surface homeostasis

## Abstract

**Objective:**

To investigate the impact of subacute preservative-containing eye drop usage on the ocular surface and to observe the clinical progression and management of medication-induced persistent corneal epithelial defects (MI-PCED).

**Methods:**

This retrospective study evaluated 60 cases (60 eyes) diagnosed with MI-PCED from January 2020 to January 2024. Data on the type, frequency, and duration of topical medication usage were collected. A multidisciplinary pharmacovigilance assessment evaluated causality, severity, and preventability of the adverse events. Upon diagnosis, the offending medications were discontinued and replaced with low-toxicity, preservative-free alternatives. For advanced epithelial decompensation with stromal involvement (Grades III and IV), bandage contact lenses (BCL) and amniotic membrane transplantation (AMT) were integrated into an exploratory step-wise management approach to promote epithelial wound healing. Treatment efficacy was evaluated using slit-lamp examination and *in vivo* confocal microscopy (IVCM).

**Results:**

The clinical manifestation of MI-PCED initiated with epithelial roughness and dry eye symptoms, ultimately progressing to persistent epithelial defects and corneal ulcers. The severity of epithelial decompensation correlated significantly with the number of concurrent medications (*P* < 0.05), administration frequency (*P* < 0.001), and medication duration (*P* < 0.05). Pharmacovigilance evaluation classified 80.0% of cases as “Probable” adverse drug reactions, with 58.3% graded as moderate (Grade 2) and 41.7% as severe (Grade 3). Following 7–30 days of targeted regenerative therapy, the cure rates were 88.57% for Grade I, 90.91% for Grade II, 77.78% for Grade III, and 80.00% for Grade IV. The overall cure rate was 86.67%.

**Conclusions:**

The severity of MI-PCED is heavily dependent on the type, subacute duration, and frequency of topical ophthalmic drug use. Management requires a flexible, step-wise strategy focused on restoring ocular surface homeostasis. This includes withdrawing toxic agents, preserving tear film stability with preservative-free tears, and strategically utilizing BCL and AMT to provide mechanical protection and biological support for severe epithelial decompensation.

## Introduction

Historically referred to as toxic keratopathy, the clinical understanding of corneal damage resulting from pharmaceutical agents has evolved. The condition is now recognized primarily as a medication-induced persistent corneal epithelial defect (MI-PCED). This pathology represents a state of chronic ocular surface failure driven by the systemic or topical administration of specific medications (1). During the initial stages, clinical manifestations may present merely as dryness, redness, or a foreign body sensation, which are frequently misattributed to drug hypersensitivity or underlying disease progression (2). However, the continuous administration of these ocular surface drugs disrupts cellular energy metabolism and impairs corneal nerve function, ultimately triggering a cascade of epithelial decompensation (3).

The widespread utilization of multi-dose eye drops causes nonspecific and cumulative damage to the conjunctiva and cornea. Preservatives in topical ophthalmic preparations, particularly benzalkonium chloride (BAC), induce significant inflammatory and apoptotic responses, leading to severe cytotoxic effects on ocular structures (4). The tear film closely adheres to the corneal epithelium and plays a crucial role in maintaining optical and metabolic stability (5). Preservatives compromise this barrier either by directly degrading the anatomical integrity of the epithelium or by destabilizing the lipid layer, which ultimately stalls normal epithelial cell migration and wound healing.

Diagnosing MI-PCED remains challenging. As the disease progresses to a state of compensatory exhaustion, pseudo-dendritic corneal lesions may appear. This frequently leads to a misdiagnosis of viral keratitis. Inappropriate antiviral therapy subsequently exacerbates epithelial toxicity, resulting in worsening defects and delayed regeneration. *In vivo* confocal microscopy (IVCM) provides invaluable insights into these corneal microstructural changes. It plays a vital role in assessing inflammatory and immune activity (6). IVCM can rapidly and non-invasively differentiate MI-PCED from infectious etiologies. Therefore, evaluating the clinical features and understanding the mechanisms of corneal epithelial decompensation hold notable research significance in the management of MI-PCED.

It is also noteworthy that combining three or more topical drugs dramatically increases the risk of epithelial breakdown. Ophthalmologists must weigh the therapeutic benefits against the potential for iatrogenic ocular surface toxicity. This study aims to investigate the effects of subacute topical medication use on ocular surface structure and function. By utilizing IVCM to evaluate clinical outcomes and corneal epithelial injury patterns, this study seeks to propose a preliminary, exploratory classification for the diagnosis and hypothesize an exploratory step-wise management approach for MI-PCED. The present work advances current knowledge by providing a detailed clinical pathological correlation of ocular surface disorders associated with medications, thereby offering new insights into the microstructural changes driving epithelial decompensation.

## Method

The experimental protocol was established in accordance with the ethical guidelines of the Helsinki Declaration. This retrospective study was reviewed and approved by the Human Ethics Committee of Xiamen University affiliated with the Xiamen Eye Center (XMYKZX-LW-2024-009). All participants provided written informed consent. From January 2020 to January 2024, a study comprising 60 eyes was conducted.

### Operational definitions and grading criteria

Currently, there is no universally accepted Grade I–IV classification for medication-induced persistent corneal epithelial defects endorsed by major ophthalmic societies. Therefore, we developed a preliminary, investigator-developed exploratory classification system based on a comprehensive evaluation using slit-lamp examination and IVCM to capture the continuous spectrum of the disease observed in our cohort. We acknowledge that this exploratory classification has not yet undergone formal validation for inter-rater reliability, intra-rater reliability, or construct validity. The grading of IVCM and slit-lamp findings was performed by two independent, masked observers to minimize bias. Importantly, these observers were independent of the investigators evaluating treatment outcomes. The variables used to define Grades I-IV were restricted strictly to morphological clinical findings (e.g., epithelial shedding, defect size, and stromal involvement). Variables relating to medication exposure (e.g., type, frequency, duration) and clinical outcomes (e.g., treatment response, cure rate) were excluded from the grading criteria and reserved exclusively for outcome assessment. However, we acknowledge the inherent risk of incorporation bias in this retrospective design, and the findings should be interpreted cautiously. Our exploratory clinical MI-PCED classification involves 4 progressive stages of epithelial decompensation:

Grade I (Mild Epithelial Decompensation): Characterized by localized or diffuse punctate shedding, rough edema, and rupture of epithelial cells.
Grade II (Proliferative Compensation): Identified by hurricane, vortex, and concentric epithelial migration patterns as the cornea attempts to heal. Spot-sheet epithelial defects with compensatory basal cell shifts.
Grade III (Compensatory Exhaustion): Marked by pseudo-dendritic or fractured persistent epithelial lesions, slit-like tissue defects, and the presence of inflammatory cells.
Grade IV (Complete Decompensation): Characterized by sheet epithelial defects, stromal melting, frank corneal ulceration, bare extracellular matrix, necrotic tissue, and liquefied cellular structures.

### Clinical evaluation and treatment

The diagnosis of MI-PCED was established through a comprehensive combination of subjective patient reports and objective clinical assessments. Baseline diagnostic evaluations included a review of clinical signs and symptoms (e.g., persistent foreign body sensation, ocular redness, and photophobia), detailed slit-lamp biomicroscopy, and ocular surface fluorescein staining to assess the morphology and extent of the epithelial defects. Tear-film integrity was quantitatively assessed utilizing non-invasive tear break-up time (TBUT) and the Schirmer I test. Furthermore, IVCM was utilized to evaluate cellular-level structural changes and rule out infectious etiologies. Baseline data regarding topical medication usage were meticulously extracted from medical records, specifically documenting the total number of concurrent medications, administration frequency, and duration of exposure prior to decompensation.

After excluding microbial infections and other contraindications, patients were instructed to discontinue all previous topical medications. Subsequently, they received intense pulsed light therapy targeting the meibomian glands, combined with warm compresses and meibomian gland massage to stabilize the lipid layer (7).

In this retrospective cohort, treatment allocation was individualized rather than strictly standardized. The treating clinician selected the interventions based on their clinical assessment prior to the formal assignment of the proposed study grades. Treatment response was assessed independently of the grading system using predefined clinical outcome measures (slit-lamp examination and fluorescein staining). Concomitant interventions were recorded where applicable. The following interventions summarize the exploratory management approach utilized:

Grades I and II: Tobramycin-dexamethasone eye ointment was applied under pressure with an overnight bandage. Autologous serum eye drops and preservative-free artificial tears were administered four times daily.
Grade III: To protect the fragile epithelium from eyelid friction and promote re-epithelialization, a therapeutic bandage contact lens (BCL) was applied continuously in conjunction with preservative-free artificial tears and autologous serum.
Grade IV: For patients presenting with sheet epithelial defects, bare stroma, or impending perforation, amniotic membrane transplantation (AMT) was performed. The amniotic membrane material utilized in this research consisted of commercially available and approved biological products, specifically Ruiji Biological Amniotic Membrane (Jiangxi, China) and Ruitai Biological Amniotic Membrane (Guangzhou, China). These materials were procured from certified tissue banks, prepared utilizing sterile cryopreservation techniques, and rigorously screened for communicable diseases under strict quality control measures. AMT was utilized to suppress local immune-inflammatory responses, provide a biological scaffold, and deliver growth factors to the compromised ocular surface.

### Therapeutic outcomes

Efficacy was assessed via slit-lamp examination and fluorescein staining. Outcomes were categorized operationally as follows:

Cured: Complete resolution of symptoms and negative corneal fluorescein staining.
Improved: Symptomatic improvement with residual punctate fluorescein staining.
Invalid: Corneal epithelial defects persisted or progressed despite treatment.

### Retrospective pharmacovigilance assessment

To systematically evaluate the relationship between topical ophthalmic medications and the development of MI-PCED, a retrospective assessment was conducted by a constituted multidisciplinary team comprising a clinical pharmacologist and two senior corneal specialists. Causality was evaluated utilizing the World Health Organization-Uppsala Monitoring Centre (WHO-UMC) system (8). The severity of the adverse drug events was graded according to the Common Terminology Criteria for Adverse Events (CTCAE) version 6.0 for eye disorders. Furthermore, preventability was assessed using the modified Schumock and Thornton criteria (9). The evaluation relied on detailed clinical histories, medication logs (including exposure duration, frequency, and preservative content), and documented clinical responses to drug withdrawal (dechallenge).

### Statistical analysis

All data was entered into an Excel spreadsheet and analyzed using SPSS statistical software version 25 (IBM, Chicago, Illinois). The normality of continuous variables was assessed utilizing the Shapiro–Wilk test. Given that the group distribution of patients was unequal and skewed, continuous data are presented as the median and interquartile range representing the 25th–75th percentiles. The non parametric Kruskal–Wallis *H* test was employed for group comparisons, followed by Dunn's *post hoc* multiple comparison tests. All tests were two tailed to capture both positive and negative unexpected effects in relation to the study hypothesis. A *P* value of less than 0.05 was considered statistically significant.

## Result

### Baseline demographic and clinical characteristics

A total of 60 patients (60 eyes) were included in this study. The baseline demographic and clinical characteristics of the cohort are summarized in Table 1. The mean age of the patients was 62.4 ± 10.5 years, comprising 28 males (46.7%) and 32 females (53.3%). Consistent with the suspected pharmacological triggers, the primary clinical indications prior to epithelial decompensation were chronic glaucoma management (43.3%), severe dry eye disease (23.3%), and postoperative prophylaxis (33.3%). The specific indications for prior surgical interventions in the postoperative group primarily included cataract surgery (20.0%), pterygium excision (8.3%), and vitreoretinal surgery (5.0%). Analysis of the comorbidity profile revealed that 26.7% of patients had co-existing diabetes mellitus, and 8.3% suffered from systemic autoimmune or inflammatory diseases. Baseline ocular surface parameters indicated compromised tear film stability across the cohort.

**Table 1:** Baseline demographic and clinical characteristics.

Characteristic	Total cohort (*N* = 60)
Age (years), Mean ± SD	62.4 ± 10.5
Sex, *n* (%)	
Male	28 (46.7%)
Female	32 (53.3%)
Primary Clinical Indication, *n* (%)	
Chronic glaucoma management	26 (43.3%)
Postoperative prophylaxis	20 (33.3%)
Severe dry eye disease	14 (23.3%)
Postoperative prophylaxis	20 (33.3%)
Cataract surgery (Phacoemulsification)	12 (20.0%)
Pterygium excision	5 (8.3%)
Vitreoretinal surgery	3 (5.0%)
Systemic Comorbidities, *n* (%)	
Diabetes Mellitus	16 (26.7%)
Autoimmune/Inflammatory disease	5 (8.3%)
Baseline Ocular Surface Parameters	
Schirmer I test (mm/5 min), Mean ± SD	4.5 ± 2.1
Tear break-up time (TBUT, s), Mean ± SD	3.2 ± 1.4

### The grade of toxic keratopathy

Our clinical MI-PCED classification involves 4 progressive stages of epithelial decompensation (Figure 1). Grade I (Mild Epithelial Decompensation) is characterized by localized or diffuse punctate shedding. Grade II (Proliferative Compensation) is identified by hurricane, vortex, and concentric epithelial migration patterns as the cornea attempts to heal. Grade III (Compensatory Exhaustion) is marked by pseudo-dendritic or fractured persistent epithelial lesions. Finally, Grade IV (Complete Decompensation) is characterized by sheet epithelial defects, stromal melting, and frank corneal ulceration. IVCM analysis confirmed varying degrees of structural failure categorized into these four grades (Table 2).

**Figure 1:**
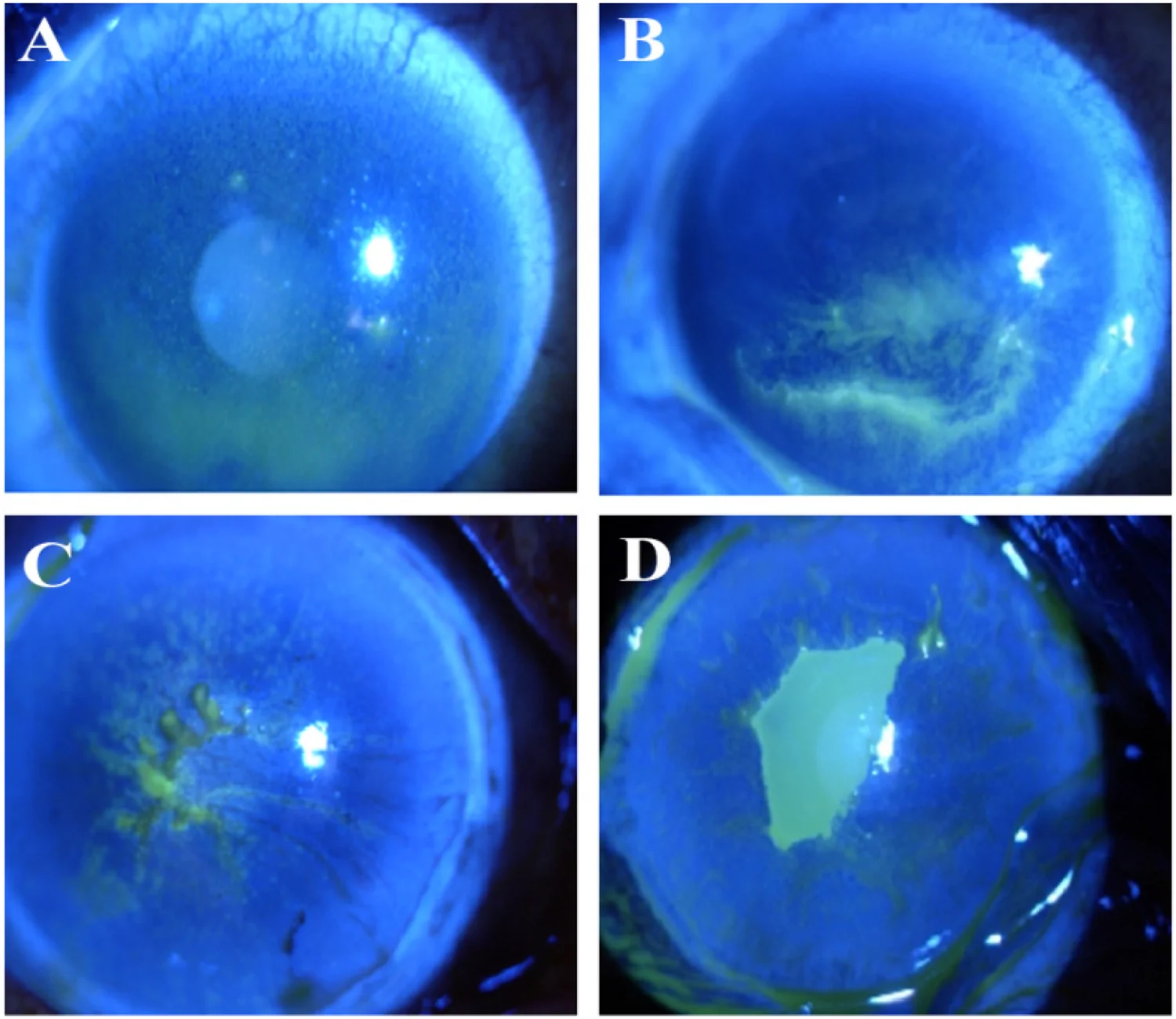
The fluorescence staining slit lamp image about different grades of the MI-PCED. **(A)** Grade I: The initial appearance is similar to a moderate dry eye epithelial dot defect, which requires medical history assessment. **(B)** Grade II: After the active period, the corneal epithelium undergoes compensatory repair which involves hyperdetachment and compensatory compensation of epithelial cells. Hurricanes, vortex, and concentric epithelial lesions characterize this period. **(C)** Grade III: If suspected drug-related corneal epithelial lesions are not treated promptly, they can progress to the third phase of the compensation deadline. **(D)** Grade IV: Compensatory limits and drug toxicity can cause corneal ulcers.

**Table 2:** The categories of MI-PCED based on IVCM characteristics and severity.

Stage	Key point
Grade I	Characterized by rough edema and rupture of epithelial cells, with inflammatory cells rarely observed (<5% of cases).
Grade II	Displayed spot sheet epithelial defects with a few detached epithelial cells, with inflammatory cells present in 15% of cases.
Grade III	Exhibited slit-like epithelial defects with a small number of inflammatory cells. Activation of stromal cells was detected in 62% of cases.
Grade IV	Presented with sheet-like defects, bare matrix, necrotic tissue, liquefied structures, and fragmented epithelial cells. Dendritic cell density significantly higher compared to other grades. Stromal cell activation and deeper structural abnormalities were observed in 91% of Grade IV cases.

### Clinical parameters and ocular surface decompensation

A detailed descriptive summary of the suspected medications, including generic names, therapeutic classes, administration frequency, duration of exposure, and specific preservative status (differentiating BAC-preserved, other-preserved, and preservative-free formulations) is provided in Table 3. The indications for the suspected medications varied widely among the cohort, primarily encompassing postoperative prophylaxis, chronic glaucoma management, and severe dry eye disease. Patients typically utilized multiple drugs in combination over an extended period. The most frequently prescribed topical agents included fluoroquinolones, preserved artificial tears, aminoglycosides, nonsteroidal anti inflammatory drugs, glucocorticoids, antivirals, and anti allergy drops.

**Table 3:** Detailed pharmacological characteristics, preservative exposure, and concomitant conditions stratified by exploratory clinical grade.

S. no.	Variable/pharmacological characteristic	Total (*N* = 60)	Grade I (*n* = 35)	Grade II (*n* = 11)	Grade III (*n* = 9)	Grade IV (*n* = 5)	Overall *P* Value
1	Primary clinical indication, *n* (%)						
	Postoperative prophylaxis	20 (33.3%)	15 (42.9%)	3 (27.3%)	2 (22.2%)	0 (0.0%)	0.142
	Chronic glaucoma management	26 (43.3%)	12 (34.3%)	5 (45.5%)	5 (55.6%)	4 (80.0%)	0.038
	Severe dry eye disease/Other	14 (23.3%)	8 (22.9%)	3 (27.3%)	2 (22.2%)	1 (20.0%)	0.895
2	Specific medication exposure by class, *n* (%)						
	Glaucoma medications						
	Latanoprost 0.005% (Sol, BAC 0.02%)	18 (30.0%)	8 (22.9%)	3 (27.3%)	4 (44.4%)	3 (60.0%)	
	Timolol maleate 0.5% (Sol, BAC 0.01%)	18 (30.0%)	6 (17.1%)	4 (36.4%)	4 (44.4%)	4 (80.0%)	
	Brimonidine tartrate 0.2% (Sol, Purite)	10 (16.7%)	4 (11.4%)	2 (18.2%)	2 (22.2%)	2 (40.0%)	
	Antibiotics/Antivirals						
	Levofloxacin 0.5% (Sol, BAC 0.005%)	24 (40.0%)	12 (34.3%)	5 (45.5%)	4 (44.4%)	3 (60.0%)	
	Tobramycin 0.3% (Sol, BAC 0.01%)	17 (28.3%)	8 (22.9%)	4 (36.4%)	3 (33.3%)	2 (40.0%)	
	Ganciclovir 0.15% (Gel, BAC 0.01%)	7 (11.7%)	3 (8.6%)	1 (9.1%)	2 (22.2%)	1 (20.0%)	
	Anti-inflammatory agents						
	Dexamethasone 0.1% (Susp, BAC 0.004%)	17 (28.3%)	7 (20.0%)	3 (27.3%)	4 (44.4%)	3 (60.0%)	
	Diclofenac sodium 0.1% (Sol, BAC 0.01%)	12 (20.0%)	5 (14.3%)	2 (18.2%)	3 (33.3%)	2 (40.0%)	
	Artificial tears/lubricants						
	Sodium Hyaluronate 0.1% (Sol, PF)	40 (66.7%)	20 (57.1%)	8 (72.7%)	7 (77.8%)	5 (100%)	
	Polyethylene Glycol 400 (Sol, Polyquad)	19 (31.7%)	10 (28.6%)	4 (36.4%)	3 (33.3%)	2 (40.0%)	
3	Preservative exposure profile						
	Concurrent multiple preparations (≥3), *n* (%)	43 (71.7%)	22 (62.9%)	8 (72.7%)	8 (88.9%)	5 (100%)	0.045
	Exposure to BAC-preserved formulations, *n* (%)	47 (78.3%)	25 (71.4%)	9 (81.8%)	8 (88.9%)	5 (100%)	0.031
	Median BAC-containing drops used concurrently	-	1 (1–2)	2 (1–3)	3 (2–4)	4 (3–5)	<0.001
	Exposure to alternative preservatives, *n* (%)	29 (48.3%)	14 (40.0%)	6 (54.5%)	5 (55.6%)	4 (80.0%)	0.215
	Strictly preservative-free regimen, *n* (%)	4 (6.7%)	4 (11.4%)	0 (0.0%)	0 (0.0%)	0 (0.0%)	0.082
	Estimated Cumulative BAC Exposure (Drops/day)	-	4 (2–8)	6 (4–10)	12 (8–15)	16 (12–20)	<0.001
4	Systemic and ocular co-morbidities, *n* (%)						
	Diabetes Mellitus	16 (26.7%)	6 (17.1%)	3 (27.3%)	4 (44.4%)	3 (60.0%)	0.042
	Autoimmune/Inflammatory disease	5 (8.3%)	2 (5.7%)	1 (9.1%)	1 (11.1%)	1 (20.0%)	0.671
	Prior ocular surgery (within 6 months)	26 (43.3%)	18 (51.4%)	4 (36.4%)	3 (33.3%)	1 (20.0%)	0.185
	Pre-existing corneal hypoesthesia	11 (18.3%)	3 (8.6%)	2 (18.2%)	3 (33.3%)	3 (60.0%)	0.012

BAC, Benzalkonium chloride; Sol, solution; Susp, suspension; PF, preservative-free; Polyquad, polyquaternium-1.

Continuous variables are presented as Median (Interquartile Range) representing the 25th–75th percentiles. Categorical variables are presented as numbers and percentages *n* (%). Overall *P* values for continuous variables were calculated using the Kruskal–Wallis *H* test. Categorical variables were analyzed using the Chi-square test or Fisher's exact test, as appropriate.

To investigate the association between topical medication burden and the severity of suspected medication-associated persistent corneal epithelial defects, clinical parameters specifically the number of concurrent medications, cumulative daily administration frequency, and duration of drug exposure were stratified across the four clinical grades. Given the skewed distribution and unequal sample sizes among the cohorts, a non parametric Kruskal–Wallis *H* test was employed. The analysis revealed a highly significant global variance across the disease severity grades for the number of concurrent medications (*H* = 19.02, *P* < 0.001), cumulative daily instillations (*H* = 13.91, *P* = 0.003), and medication duration (*H* = 15.70, *P* = 0.001).

Subsequent *post hoc* evaluation utilizing Dunn's test with Bonferroni adjustment elucidated specific differences between groups. A clear dose response relationship was observed regarding polypharmacy. The median number of concurrent drugs ranged from 3 in Grade I up to 5 in Grade IV. Patients exhibiting complete epithelial decompensation (Grade IV) required significantly more concurrent drug types compared to the Grade I (*P* = 0.0008) and Grade II (*P* = 0.0010) cohorts. Furthermore, advanced epithelial decompensation was tightly linked to instillation frequency. Grade IV and Grade III patients demonstrated a marked elevation in cumulative daily instillations compared to Grade II patients (*P* = 0.010 and *P* = 0.033, respectively). Prolonged drug exposure also drove pathological progression, as Grade III patients exhibited significantly longer medication durations than the Grade I cohort (*P* = 0.004). These findings underscore that profound ocular surface toxicity is critically dependent on the compounding effects of multi drug exposure, high instillation frequency, and extended treatment duration (Table 4 and Figure 2).

**Table 4:** Eye drops usage, treatment efficacy, and *in vivo* confocal microscopy cell count of different grade corneal epithelial lesions.

Variable	Grade I (*n* = 35)	Grade II (*n* = 11)	Grade III (*n* = 9)	Grade IV (*n* = 5)	Overall *P* value
Number of concurrent medications (Median, IQR)	3 (3–3)	3 (2.5–3)	4 (3–4)	5 (5–5)^a^^,^^b^	<0.001
Cumulative frequency (times per day) (Median, IQR)	12 (10.5–13.5)	9 (8–12)	15 (12–16)^c^	16 (15–20)^d^	<0.01
Medication duration (days) (Median, IQR)	15 (14–19)	20 (15.5–24.5)	25 (21–30)^e^	24 (22–30)	<0.01
Corneal epithelial cell count (Mean ± SD)	4,024 ± 452	2,739 ± 355	1,160 ± 142	558 ± 186	<0.001
Therapeutic outcome					>0.05
Cure	31	10	7	4	
Improve	4	1	2	1	
Invalid	0	0	0	0	

Continuous clinical data are presented as Median (Interquartile Range) representing the 25th–75th percentiles. Overall *P* values were calculated utilizing the Kruskal–Wallis *H* test. *Post hoc* Dunn's tests with Bonferroni correction yielded the following significant pairwise differences.

a *P* = 0.0008 compared to Grade I.

b *P* = 0.0010 compared to Grade II.

c *P* = 0.0331 compared to Grade II.

d *P* = 0.0101 compared to Grade II.

e *P* = 0.0044 compared to Grade I.

**Figure 2:**
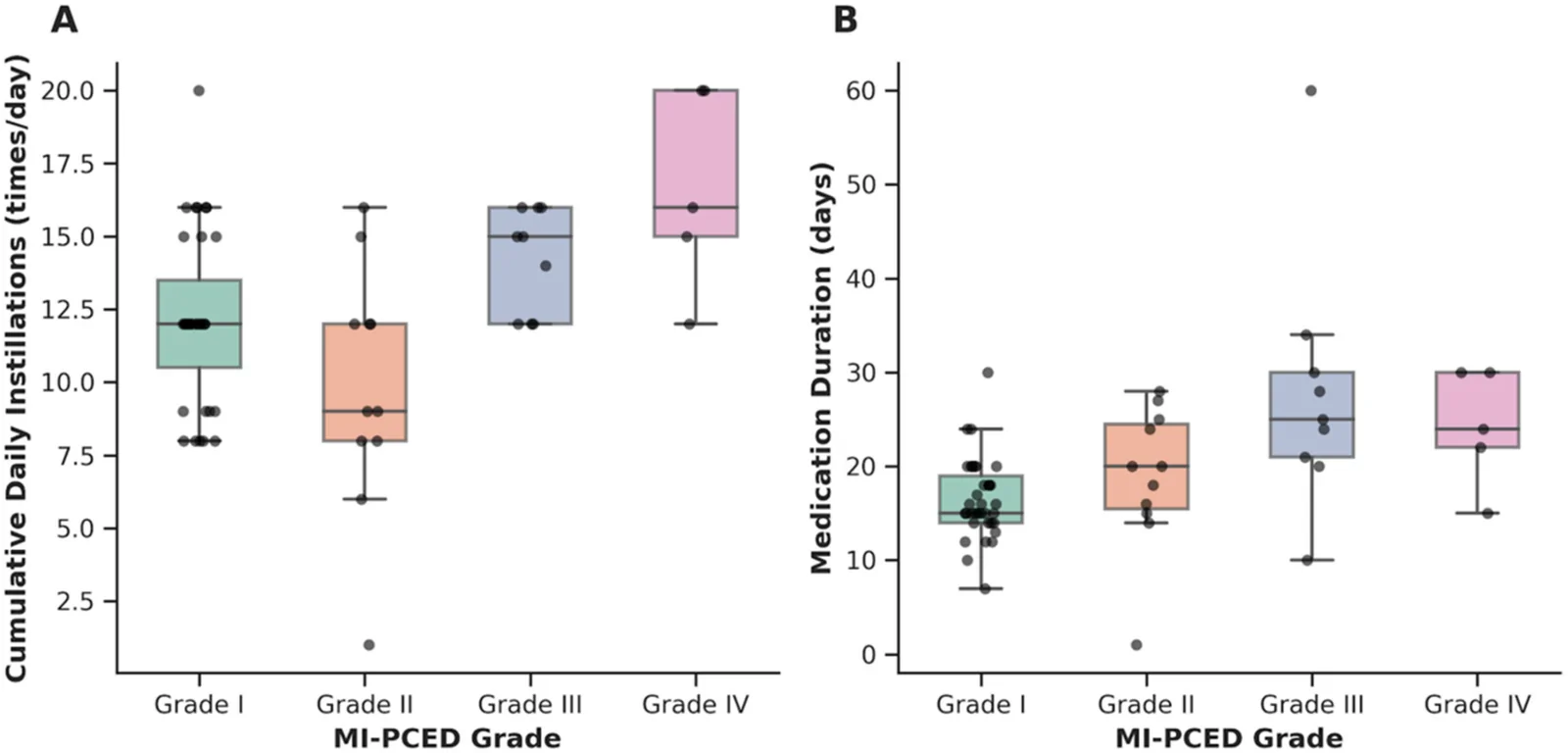
The impact of administration frequency and medication duration on the severity of medication-induced persistent corneal epithelial defects (MI-PCED). **(A)** Boxplot illustrating the cumulative daily instillations (times per day) stratified by MI-PCED grade. Patients in the severe decompensation stages (Grades III and IV) exhibited a marked elevation in instillation frequencies compared to Grade II. **(B)** Boxplot depicting the duration of medication exposure (days) across different clinical grades. Advanced epithelial exhaustion (Grade III) was associated with significantly prolonged drug exposure compared to the mild cohort (Grade I). For both panels, horizontal lines represent the median, boxes represent the interquartile range (25th–75th percentiles), and whiskers indicate the minimum and maximum values (excluding outliers). Individual data points are jittered and overlaid to demonstrate the true distribution of the raw data.

### Therapeutic outcomes

Following 7 to 30 days of regenerative treatment, the cure rates were evaluated. The cure rate was 88.57% (31/35) for Grade I, 90.91% (10/11) for Grade II, 77.78% (7/9) for Grade III, and 80.00% (4/5) for Grade IV. The overall cure rate across all grades was 86.67%. Treatment duration increased significantly in tandem with disease severity. Long term follow up (more than 6 months) revealed that once complete epithelial homeostasis was restored, no recurrences were observed. This indicates an excellent long term prognosis following appropriate biological management.

### Corneal confocal microscopy image feature

IVCM revealed the microstructural reality of epithelial failure. Grade I showed rough, edematous, and ruptured surface cells. Grade II exhibited spot-sheet epithelial defects with compensatory basal cell shifts. Grade III demonstrated slit-like tissue defects and the presence of inflammatory cells. This marked the exhaustion of the regenerative compartment. Grade IV displayed massive local defects, bare extracellular matrix, necrotic tissue, liquefied cellular structures, and highly fragmented cells.

Higher grades correlated with a complete loss of epithelial hierarchy and organization. Grades I and II did not involve the stroma. In contrast, Grades III and IV exhibited aggressive stromal cell activation. Inflammatory cells peaked in Grade III, while dendritic cells reached their highest density in Grade IV. No significant endothelial changes were observed across the grades (Figure 3).

**Figure 3:**
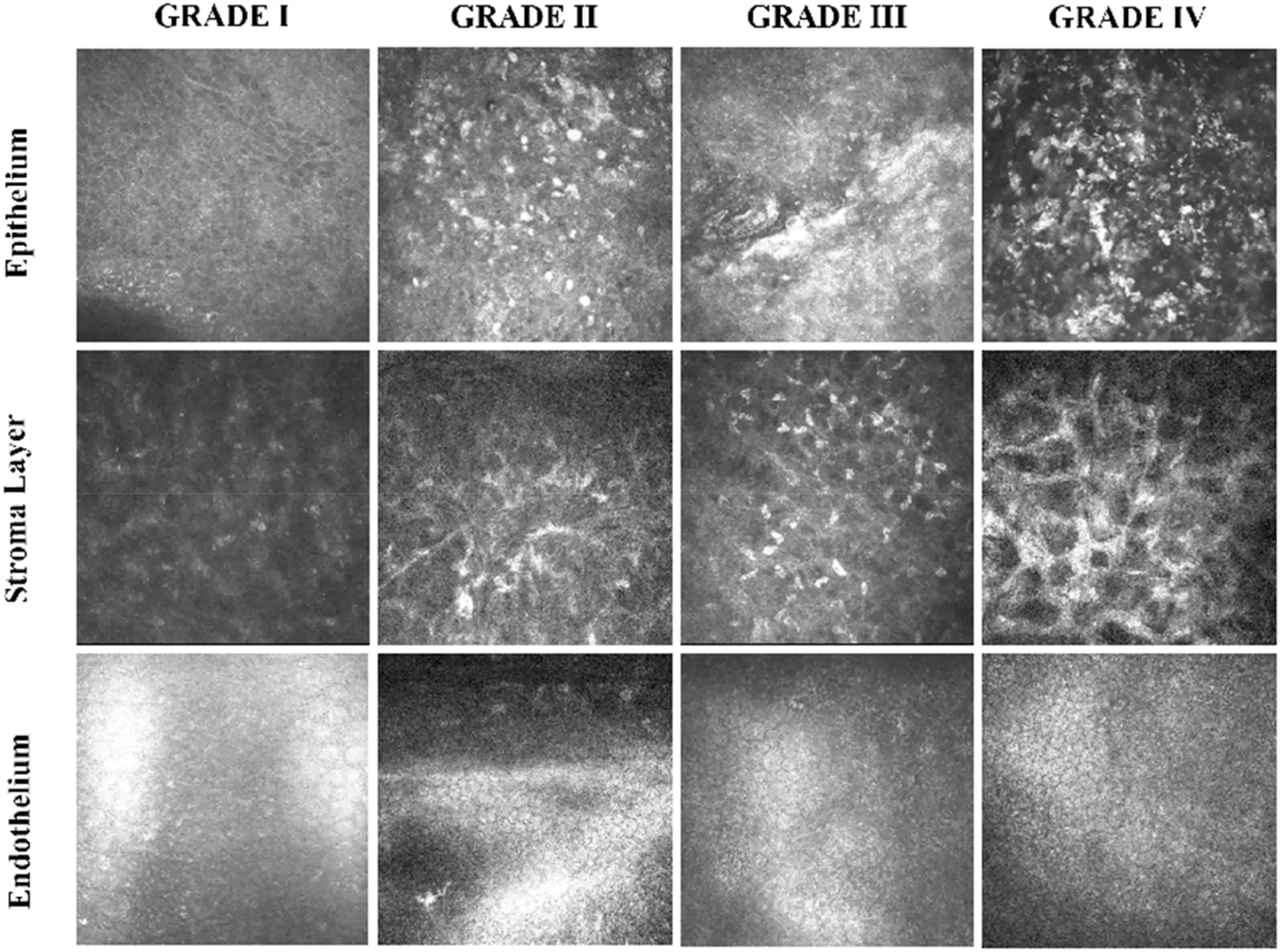
IVCM image features across different MI-PCED grades. When observing the microstructure of the cornea under IVCM, there was no noticeable inflammatory or immune response in the initial stages of MI-PCED. However, as the grade ascended, epithelial decompensation became more prevalent. We noted that Grade III MI-PCED is a critical period of compensation. Ultimately, in Grade IV, epithelial regeneration is completely decompensated, and numerous necrotic tissues and liquefied structures are present in the epithelial layer, along with active infiltration of Langerhans cells between the layers. Although there was no significant damage to the matrix and endothelial surface in different grades, the superficial matrix layer could be activated in high-level MI-PCED.

### Causality, severity, and preventability of adverse drug events

A retrospective multidisciplinary evaluation of the 60 cases using the WHO-UMC causality assessment system revealed that 48 cases (80.0%) were classified as “Probable” adverse drug reactions (detailed patient-level evaluation data are provided in Supplementary Table S1). In these cases, there was a clear temporal sequence between medication administration and corneal decompensation, and the epithelial defects demonstrated significant clinical improvement (86.67% overall cure rate) upon drug withdrawal and substitution with preservative-free alternatives. Twelve cases (20.0%) were classified as “Possible,” as underlying severe dry eye disease or diabetic co-morbidities (present in 26.7% of the total cohort) provided alternative, though not exhaustive, pathophysiological explanations for the persistent defects. No cases were classified as “Certain” due to the ethical contraindication of performing a positive rechallenge in patients with severe ocular surface toxicity. Regarding severity (CTCAE v6.0), the 35 cases (58.3%) corresponding to exploratory Grade I were classified as Grade 2 (moderate) adverse events. The remaining 25 cases (41.7%), encompassing exploratory Grades II–IV with partial to complete stromal involvement and requirement for advanced interventions (BCL or AMT), were classified as Grade 3 (severe) adverse events. Preventability assessment indicated that a vast majority of cases were “Probably Preventable,” primarily by minimizing polypharmacy (defined as concurrent use of 3 or more preserved drops, seen in 71.7% of cases) and reducing cumulative daily BAC exposure earlier in the clinical course.

## Discussion

Despite advances in ophthalmic diagnostics and therapeutics, ocular surface disease associated with medications remains a persistent clinical challenge. Eye drops, though commonly used to treat eye diseases, can cause serious side effects and toxic damage to the ocular surface (10). Chemical injuries and prolonged exposure to iatrogenic agents are dominant drivers of persistent ocular surface defects (11). Eye drops are essential for managing ophthalmic conditions. However, their inappropriate or prolonged application severely disrupts the regenerative capacity of the corneal epithelium, leading to MI-PCED (12). Rather than simple acute chemical burns, these presentations are characterized by a chronic failure of wound healing, chronic conjunctival papillary hyperplasia, and ultimate stem cell or basal cell exhaustion (13). Data from this study suggest an association between topical drug exposure and the disruption of ocular surface homeostasis, which may contribute to continuous epithelial disorder. Given the retrospective nature of this study, a comprehensive assessment of alternative explanations (e.g., underlying dry eye disease severity, neurotrophic keratopathy, or concurrent systemic diseases) could not be fully systematically controlled. Therefore, causal language is avoided, and these clinical presentations are more appropriately described as suspected medication-associated epithelial defects. The risk of corneal toxicity increased proportionally with the number of agents administered and the extended duration of treatment cycles. While local ophthalmic treatment of the corneal epithelium at appropriate concentrations and frequency can be tolerated, it often causes discomfort to patients after the stability of the tear film is destroyed. We observed the different grades of MI-PCED through IVCM and suggests that the peak of inflammatory response occurs when the compensatory deadline of epithelial function is reached in Grade III. At Grade IV, the release of corneal antigens and extensive stromal activation reflected a heightened immune-inflammatory state. In addition, as the grade increases, epithelial basal cells decline more. Using IVCM, we can identify unstable tear film and non-infectious corneal ulcers in dry eyes, which can help diagnose MI-PCED.

Progressive multi-drug exposure induces extensive ocular surface damage, ranging from mild superficial punctate lesions to severe corneal melting. Direct cytotoxicity and chronic inflammation can permanently alter ocular tissue structure (14). Various active pharmaceutical ingredients contribute significantly to this pathology (15). Aminoglycosides and nonsteroidal anti-inflammatory drugs frequently induce punctate keratitis and conjunctival edema (16). Fluoroquinolones promote matrix metalloproteinase expression and increase the risk of corneal perforation, while extended glucocorticoid application actively delays wound healing (17). Furthermore, topical anesthetics exert profound direct cytotoxicity, impairing cell migration and reducing mitochondrial viability. Prolonged use of these therapeutic agents eventually triggers a cascade of toxic reactions. These include filamentous keratitis and pseudo-dendritic lesions that severely compromise vision (18).

Preservatives represent another critical factor in ocular surface toxicity. They improve drug stability but exhibit massive cytotoxic potential during long-term use. BAC is the most ubiquitous and toxic preservative (19). BAC severely destabilizes the tear film lipid layer and disrupts the epithelial barrier. It triggers robust immune-inflammatory responses through oxidative stress and the release of proinflammatory cytokines (20, 21). Apoptosis and deeper degenerative changes occur when preservative concentrations exceed the epithelial compensatory threshold. Consequently, the incidence of MI-PCED is significantly higher with preserved formulations compared to preservative-free alternatives. Patients with chronic surface disease, prior ocular surgery, or extended contact lens wear are particularly vulnerable to these destructive effects (22). Distinguishing the specific toxicity associated with preservatives from that of the APIs in this patient cohort is challenging, as these components often synergistically exacerbate epithelial breakdown during concurrent multi-drug administration.

Current treatment for MI-PCED involves the immediate discontinuation of the offending drug. Clinicians then initiate preservative-free artificial tears to halt progressive corneal damage (23). Compared with standard artificial tears, autologous serum eye drops have been shown to better restore the morphological and functional integrity of the epithelium (24). From a clinical perspective, determining the optimal treatment strategy requires a comprehensive assessment of clinical signs and symptoms, the current state of inflammatory immunity, and the ability of epithelial cells to repair on the ocular surface (25). When MI-PCED progresses beyond the compensatory limits of the corneal epithelium (Grades III and IV), simply withdrawing the offending medications and providing artificial tears is often insufficient (26). In our study, we introduced BCL and AMT to address severe ocular surface failure. BCLs act as a mechanical shield, protecting the actively migrating basal epithelial cells from the shearing forces of the eyelids during blinking (27). By maintaining a localized reservoir of tears and autologous serum over the compromised cornea, BCLs stabilize the microenvironment and accelerate wound closure in pseudo-dendritic or fractured epithelial lesions (28).

For Grade IV MI-PCED, where deep stromal infiltration, necrosis, and high dendritic cell density denote a severe immune-inflammatory state, AMT proved highly effective. The amniotic membrane contains abundant anti-inflammatory cytokines, protease inhibitors, and growth factors that directly counteract the cytotoxic and fibrotic pathways triggered by prolonged drug toxicity (29–31). Furthermore, it serves as a critical biological scaffold that promotes epithelial cell adhesion and differentiation while preventing deeper stromal melting (32, 33). Our IVCM findings which highlighted severe structural liquefaction and cellular loss at this stage underscore the necessity of the biological intervention provided by AMT to restore ocular surface anatomy.

Even after recovery, it is essential to maintain continuous and dynamic management. Apart from temporary defects in the epithelial layer, the corneal structure can also produce immune complex deposition and stromal remodeling, which could be the reason for disease progression and permanent damage to the corneal structure (34). Our findings highlight the importance of accurate etiological diagnosis, rational drug use, and minimizing multi-drug exposure. It is recommended to avoid excessive use of multiple medications and to limit the frequency and duration of drug intake. In case of MI-PCED diagnosis or suspicion, discontinue all topical eye drugs. The use of preservative-free or low-preservative eye drops, in combination with agents that promote epithelial repair, remains a cornerstone of MI-PCED management (Figure 4).

**Figure 4:**
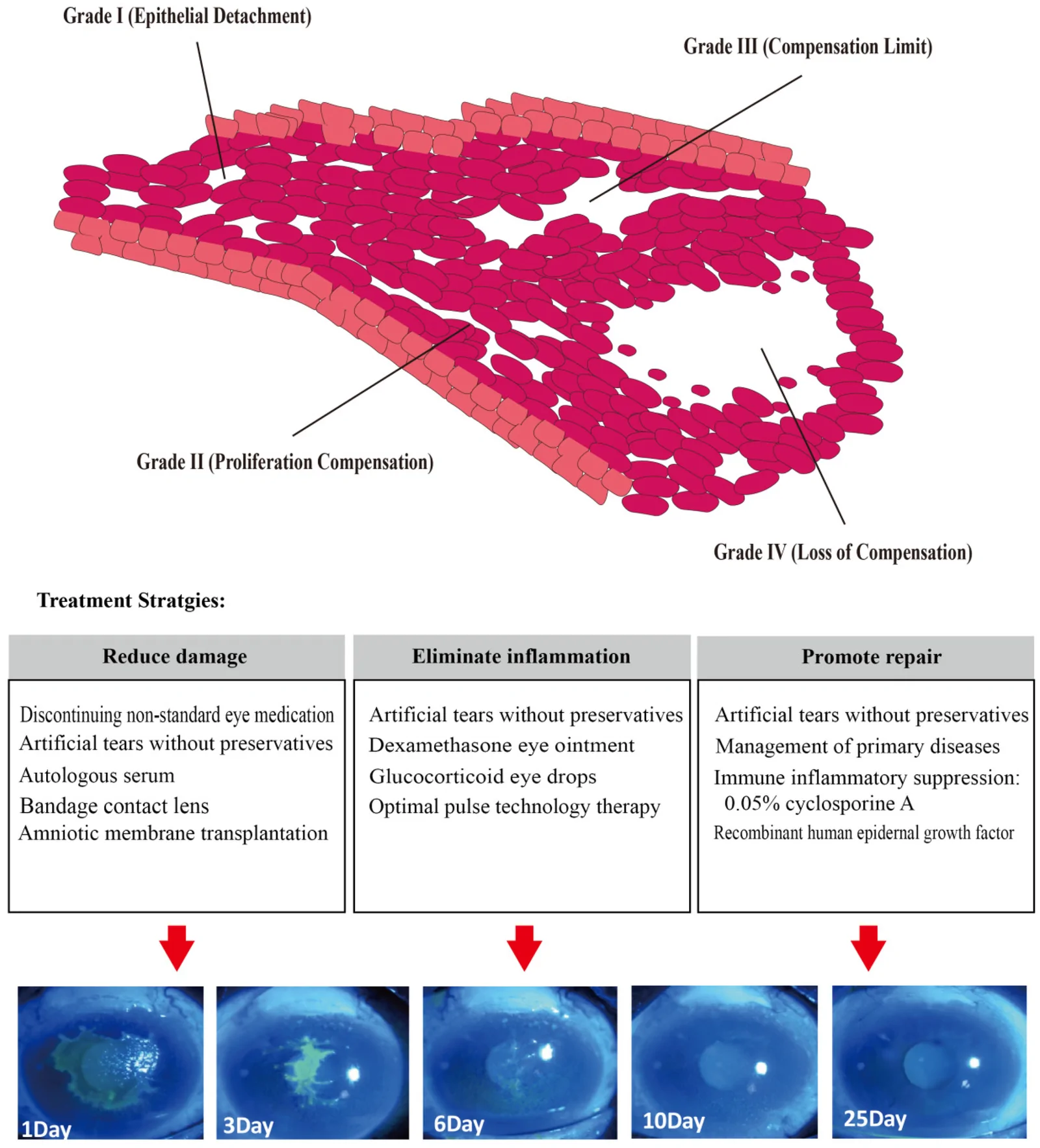
Treatment strategies for MI-PCED in different ocular surface states. For decompensated corneal epithelium, drugs should be stopped to promote the repair of angular conjunctival damage. Moderate anti-inflammatory treatment should be given for ocular surface inflammation. Throughout the course, artificial tears without preservatives should be used to maintain the stability of the tear film and provide conditions for epithelial repair. In addition, during the process of epithelial repair, the immune-inflammatory response of the cornea should be noted, and cyclosporin A used in local immunotherapy is also an important measure in the management of ocular surface homeostasis.

Several limitations of the current study warrant consideration. First, the retrospective design and unequal sample sizes, notably within severe Grade III and IV cohorts, introduce potential selection bias and statistical limitations. Furthermore, the Grade I–IV classification utilized in this study is a preliminary, investigator-developed exploratory tool. Because formal validation could not be performed using this retrospective dataset, prospective, multicenter validation, including rigorous assessments of inter-rater and intra-rater reliability is required before this classification system or its associated management algorithm can be generalized or recommended for routine clinical use. Second, heterogeneous patient exposure to varied topical medications and preservative concentrations precludes the precise isolation of individual pathophysiological triggers. Additionally, while formal causality, severity, and preventability evaluations were conducted using established frameworks (WHO-UMC, CTCAE version 6.0, and modified Schumock and Thornton criteria), these assessments were inherently constrained by the study's retrospective nature. Because the necessary granular information was not prospectively collected, definitive causality (e.g., via a positive drug rechallenge) could not be ethically or practically confirmed. Additionally, specific socio-economic and lifestyle variables, such as educational status, per-capita income, occupation, and substance-use/addiction status, are not routinely captured in standard ophthalmic outpatient electronic medical records and were therefore unavailable for retrospective extraction and analysis. We explicitly state that our proposed exploratory Grade I-IV classification is intended to complement, rather than replace, these standardized pharmacovigilance assessments. Third, the concurrent administration of severity stratified interventions confounds the delineation of independent therapeutic efficacies. Additionally, while identifying independent predictors of treatment outcomes via multivariable logistic or Cox proportional hazards regression would provide substantial clinical value, our dataset lacked sufficient statistical power for such modeling. With a high overall cure rate of 86.67% (52 cured vs. 8 not cured), incorporating multiple covariates into a predictive model would risk severe mathematical bias and overfitting. Consequently, we relied on univariable and descriptive analyses. Fourth, a restricted observation period of 4–12 months compromises the comprehensive assessment of long term clinical outcomes. Consequently, future prospective investigations utilizing larger independent cohorts and strictly controlled variables remain essential for rigorous clinical grading system validation and targeted MI-PCED therapeutic refinement.

## Conclusion

The prolonged and frequent use of topical ophthalmic medications can trigger severe epithelial decompensation. This leads to MI-PCED. Beyond temporary cellular shedding, the cornea undergoes profound immune-inflammatory activation, neural impairment, and fibrotic remodeling. These factors stall the natural wound healing process. Our retrospective pharmacovigilance assessment underscores that the vast majority of these adverse events are “Probable” and largely preventable. In clinical practice, mitigating multi-drug exposure and minimizing preservative loads are paramount. We propose that the management of MI-PCED may benefit from an exploratory graded and personalized approach. Clinicians should consider utilizing preservative-free agents to restore homeostasis, and may explore deploying mechanical scaffolds like BCL or biological scaffolds like AMT when the epithelial regenerative capacity is exhausted. In the absence of multivariable modeling and prospective evaluation, this step-wise management algorithm should currently be viewed as a clinical hypothesis requiring future independent validation.

## Data Availability

The original contributions presented in the study are included in the article/Supplementary Material, further inquiries can be directed to the corresponding authors.
