# Supplementary material for: Clinical features and management of medication-induced persistent corneal epithelial defects due to topical ophthalmic medications
Source: Front Med (Lausanne). 2026 Sep 25;13:1928830. doi: 10.3389/fmed.2026.1928830 (PMC13649420; doi:10.3389/fmed.2026.1928830)
Supplement: Supplementary file 1 [file Table1.docx]

Supplementary Table 1 Patient-Level Pharmacovigilance Assessment of Suspected Topical Ophthalmic Medications, Causality, Severity, and Preventability

| **Patient** | **Age**  **(years)** | **Sex** | **MI-PCED Grade** | **Primary Indication** | **Suspected Topical Medications (Conc., Preservative)** | **Exposure Duration (days)** | **Concurrent Meds (n)** | **Causality (WHO-UMC)** | **Severity (CTCAE v6.0)** | **Preventability** | **Outcome** |
| --- | --- | --- | --- | --- | --- | --- | --- | --- | --- | --- | --- |
| P01 | 68 | F | Grade I | Postoperative | Sodium Hyaluronate 0.1% (PF), Autologous Serum (PF) | 14 | 2 | Probable | Grade 2 | Not Preventable | Cured |
| P02 | 72 | M | Grade I | Glaucoma | Sodium Hyaluronate 0.1% (PF), Autologous Serum (PF) | 16 | 2 | Probable | Grade 2 | Definitely Preventable | Cured |
| P03 | 65 | F | Grade II | Glaucoma | Latanoprost (BAC), Brimonidine (Purite), Dexamethasone (BAC) | 20 | 3 | Possible | Grade 3 | Probably Preventable | Cured |
| P04 | 55 | F | Grade III | Dry Eye | Tobramycin (BAC), Diclofenac (BAC), PEG 400 (Polyquad) | 25 | 3 | Probable | Grade 3 | Probably Preventable | Cured |
| P05 | 77 | M | Grade IV | Glaucoma | Timolol (BAC), Latanoprost (BAC), Levofloxacin (BAC), Dex (BAC) | 22 | 4 | Probable | Grade 3 | Definitely Preventable | Cured |
| P06 | 61 | M | Grade I | Postoperative | Sodium Hyaluronate 0.1% (PF), Autologous Serum (PF) | 15 | 2 | Probable | Grade 2 | Probably Preventable | Cured |
| P07 | 58 | F | Grade I | Dry Eye | Sodium Hyaluronate 0.1% (PF), Autologous Serum (PF) | 12 | 2 | Probable | Grade 2 | Not Preventable | Cured |
| P08 | 70 | M | Grade I | Glaucoma | Latanoprost 0.005% (BAC), Brimonidine 0.2% (Purite), PEG 400 (Polyquad) | 14 | 3 | Probable | Grade 2 | Probably Preventable | Cured |
| P09 | 63 | F | Grade I | Postoperative | Tobramycin 0.3% (BAC), PEG 400 (Polyquad), PEG 400 (Polyquad) | 18 | 3 | Probable | Grade 2 | Not Preventable | Cured |
| P10 | 66 | M | Grade I | Postoperative | Levofloxacin 0.5% (BAC), Dexamethasone 0.1% (BAC), Sodium Hyaluronate 0.1% (PF) | 16 | 3 | Probable | Grade 2 | Probably Preventable | Cured |
| P11 | 74 | F | Grade I | Glaucoma | Timolol 0.5% (BAC), PEG 400 (Polyquad), PEG 400 (Polyquad) | 19 | 3 | Probable | Grade 2 | Probably Preventable | Cured |
| P12 | 69 | M | Grade I | Glaucoma | Latanoprost 0.005% (BAC), Timolol 0.5% (BAC), Sodium Hyaluronate 0.1% (PF) | 14 | 3 | Probable | Grade 2 | Definitely Preventable | Cured |
| P13 | 59 | F | Grade I | Postoperative | Levofloxacin 0.5% (BAC), Diclofenac (BAC), PEG 400 (Polyquad) | 11 | 3 | Probable | Grade 2 | Probably Preventable | Cured |
| P14 | 52 | F | Grade I | Dry Eye | Tobramycin 0.3% (BAC), Sodium Hyaluronate 0.1% (PF), PEG 400 (Polyquad) | 15 | 3 | Probable | Grade 2 | Not Preventable | Cured |
| P15 | 67 | M | Grade I | Postoperative | Ganciclovir 0.15% (BAC), Levofloxacin 0.5% (BAC), PEG 400 (Polyquad) | 20 | 3 | Probable | Grade 2 | Probably Preventable | Cured |
| P16 | 71 | M | Grade I | Glaucoma | Brimonidine 0.2% (Purite), Timolol 0.5% (BAC), PEG 400 (Polyquad) | 17 | 3 | Probable | Grade 2 | Probably Preventable | Cured |
| P17 | 64 | F | Grade I | Postoperative | Levofloxacin 0.5% (BAC), Dexamethasone 0.1% (BAC), PEG 400 (Polyquad) | 13 | 3 | Probable | Grade 2 | Probably Preventable | Cured |
| P18 | 75 | M | Grade I | Glaucoma | Latanoprost 0.005% (BAC), Sodium Hyaluronate 0.1% (PF), PEG 400 (Polyquad) | 15 | 3 | Probable | Grade 2 | Not Preventable | Cured |
| P19 | 60 | F | Grade I | Postoperative | Tobramycin 0.3% (BAC), Dexamethasone 0.1% (BAC), PEG 400 (Polyquad) | 14 | 3 | Probable | Grade 2 | Probably Preventable | Cured |
| P20 | 54 | F | Grade I | Dry Eye | Diclofenac (BAC), PEG 400 (Polyquad), PEG 400 (Polyquad) | 10 | 3 | Possible | Grade 2 | Not Preventable | Cured |
| P21 | 68 | M | Grade I | Postoperative | Levofloxacin 0.5% (BAC), Sodium Hyaluronate 0.1% (PF), PEG 400 (Polyquad) | 16 | 3 | Possible | Grade 2 | Not Preventable | Cured |
| P22 | 73 | M | Grade I | Glaucoma | Timolol 0.5% (BAC), Dexamethasone 0.1% (BAC), Brimonidine 0.2% (Purite) | 19 | 3 | Probable | Grade 2 | Definitely Preventable | Cured |
| P23 | 57 | F | Grade I | Dry Eye | Sodium Hyaluronate 0.1% (PF), Ganciclovir 0.15% (BAC), PEG 400 (Polyquad) | 15 | 3 | Possible | Grade 2 | Not Preventable | Cured |
| P24 | 62 | M | Grade I | Postoperative | Levofloxacin 0.5% (BAC), Diclofenac (BAC), PEG 400 (Polyquad) | 14 | 3 | Probable | Grade 2 | Probably Preventable | Cured |
| P25 | 76 | F | Grade I | Glaucoma | Latanoprost 0.005% (BAC), PEG 400 (Polyquad), PEG 400 (Polyquad) | 17 | 3 | Possible | Grade 2 | Not Preventable | Cured |
| P26 | 65 | F | Grade I | Postoperative | Tobramycin 0.3% (BAC), Dexamethasone 0.1% (BAC), Sodium Hyaluronate 0.1% (PF) | 12 | 3 | Probable | Grade 2 | Probably Preventable | Cured |
| P27 | 69 | M | Grade I | Postoperative | Levofloxacin 0.5% (BAC), Timolol 0.5% (BAC), PEG 400 (Polyquad) | 15 | 3 | Probable | Grade 2 | Probably Preventable | Cured |
| P28 | 51 | F | Grade I | Dry Eye | PEG 400 (Polyquad), Diclofenac (BAC), Sodium Hyaluronate 0.1% (PF) | 16 | 3 | Possible | Grade 2 | Probably Preventable | Cured |
| P29 | 71 | M | Grade I | Glaucoma | Brimonidine 0.2% (Purite), Latanoprost 0.005% (BAC), PEG 400 (Polyquad) | 18 | 3 | Probable | Grade 2 | Probably Preventable | Cured |
| P30 | 59 | F | Grade I | Postoperative | Levofloxacin 0.5% (BAC), Dexamethasone 0.1% (BAC) | 11 | 2 | Probable | Grade 2 | Probably Preventable | Cured |
| P31 | 74 | F | Grade I | Glaucoma | Timolol 0.5% (BAC), Sodium Hyaluronate 0.1% (PF) | 14 | 2 | Possible | Grade 2 | Not Preventable | Cured |
| P32 | 48 | F | Grade I | Dry Eye | Ganciclovir 0.15% (BAC), PEG 400 (Polyquad) | 15 | 2 | Probable | Grade 2 | Not Preventable | Cured |
| P33 | 67 | M | Grade I | Postoperative | Tobramycin 0.3% (BAC), Diclofenac (BAC) | 13 | 2 | Probable | Grade 2 | Probably Preventable | Cured |
| P34 | 78 | M | Grade I | Glaucoma | Latanoprost 0.005% (BAC), Timolol 0.5% (BAC) | 19 | 2 | Probable | Grade 2 | Definitely Preventable | Cured |
| P35 | 61 | F | Grade I | Postoperative | Levofloxacin 0.5% (BAC), PEG 400 (Polyquad) | 16 | 2 | Possible | Grade 2 | Not Preventable | Improved |
| P36 | 55 | F | Grade I | Dry Eye | Sodium Hyaluronate 0.1% (PF), Diclofenac (BAC) | 17 | 2 | Probable | Grade 2 | Probably Preventable | Improved |
| P37 | 72 | M | Grade I | Glaucoma | Brimonidine 0.2% (Purite), Timolol 0.5% (BAC) | 14 | 2 | Probable | Grade 2 | Probably Preventable | Improved |
| P38 | 66 | F | Grade I | Postoperative | Tobramycin 0.3% (BAC), Dexamethasone 0.1% (BAC) | 12 | 2 | Probable | Grade 2 | Probably Preventable | Improved |
| P39 | 75 | M | Grade II | Glaucoma | Latanoprost 0.005% (BAC), Timolol 0.5% (BAC), Sodium Hyaluronate 0.1% (PF) | 22 | 3 | Probable | Grade 3 | Definitely Preventable | Cured |
| P40 | 63 | F | Grade II | Postoperative | Levofloxacin 0.5% (BAC), Dexamethasone 0.1% (BAC), Diclofenac (BAC) | 18 | 3 | Probable | Grade 3 | Probably Preventable | Cured |
| P41 | 58 | F | Grade II | Dry Eye | Tobramycin 0.3% (BAC), PEG 400 (Polyquad), Sodium Hyaluronate 0.1% (PF) | 21 | 3 | Possible | Grade 3 | Probably Preventable | Cured |
| P42 | 70 | M | Grade II | Glaucoma | Brimonidine 0.2% (Purite), Latanoprost 0.005% (BAC), Dexamethasone 0.1% (BAC) | 24 | 3 | Probable | Grade 3 | Definitely Preventable | Cured |
| P43 | 64 | M | Grade II | Postoperative | Levofloxacin 0.5% (BAC), Tobramycin 0.3% (BAC), Sodium Hyaluronate 0.1% (PF) | 16 | 3 | Probable | Grade 3 | Probably Preventable | Cured |
| P44 | 76 | F | Grade II | Glaucoma | Timolol 0.5% (BAC), Diclofenac (BAC), PEG 400 (Polyquad) | 20 | 3 | Probable | Grade 3 | Probably Preventable | Cured |
| P45 | 53 | F | Grade II | Dry Eye | Ganciclovir 0.15% (BAC), Levofloxacin 0.5% (BAC), Sodium Hyaluronate 0.1% (PF) | 19 | 3 | Possible | Grade 3 | Probably Preventable | Cured |
| P46 | 69 | M | Grade II | Glaucoma | Latanoprost 0.005% (BAC), Timolol 0.5% (BAC), Brimonidine 0.2% (Purite) | 26 | 3 | Probable | Grade 3 | Definitely Preventable | Cured |
| P47 | 62 | F | Grade II | Postoperative | Tobramycin 0.3% (BAC), Dexamethasone 0.1% (BAC), Diclofenac (BAC) | 17 | 3 | Probable | Grade 3 | Probably Preventable | Cured |
| P48 | 74 | M | Grade II | Glaucoma | Timolol 0.5% (BAC), Latanoprost 0.005% (BAC), PEG 400 (Polyquad) | 23 | 3 | Probable | Grade 3 | Definitely Preventable | Improved |
| P49 | 71 | M | Grade III | Glaucoma | Latanoprost (BAC), Timolol (BAC), Brimonidine (Purite), Sodium Hyaluronate (PF) | 28 | 4 | Probable | Grade 3 | Definitely Preventable | Cured |
| P50 | 65 | F | Grade III | Postoperative | Levofloxacin (BAC), Dexamethasone (BAC), Diclofenac (BAC), PEG 400 (Polyquad) | 22 | 4 | Probable | Grade 3 | Definitely Preventable | Cured |
| P51 | 79 | M | Grade III | Glaucoma | Timolol (BAC), Brimonidine (Purite), Dexamethasone (BAC), Tobramycin (BAC) | 26 | 4 | Probable | Grade 3 | Definitely Preventable | Cured |
| P52 | 56 | F | Grade III | Dry Eye | Ganciclovir (BAC), Levofloxacin (BAC), Diclofenac (BAC), Sodium Hyaluronate (PF) | 24 | 4 | Possible | Grade 3 | Probably Preventable | Cured |
| P53 | 73 | M | Grade III | Glaucoma | Latanoprost (BAC), Timolol (BAC), Diclofenac (BAC), PEG 400 (Polyquad) | 30 | 4 | Probable | Grade 3 | Definitely Preventable | Cured |
| P54 | 60 | F | Grade III | Postoperative | Tobramycin (BAC), Levofloxacin (BAC), Dexamethasone (BAC), Sodium Hyaluronate (PF) | 21 | 4 | Probable | Grade 3 | Definitely Preventable | Cured |
| P55 | 78 | M | Grade III | Glaucoma | Timolol (BAC), Latanoprost (BAC), Brimonidine (Purite), Dexamethasone (BAC) | 27 | 4 | Probable | Grade 3 | Definitely Preventable | Improved |
| P56 | 68 | F | Grade III | Glaucoma | Latanoprost (BAC), Timolol (BAC), Tobramycin (BAC), PEG 400 (Polyquad) | 25 | 4 | Probable | Grade 3 | Definitely Preventable | Improved |
| P57 | 81 | M | Grade IV | Glaucoma | Timolol (BAC), Latanoprost (BAC), Brimonidine (Purite), Dex (BAC), Hyaluronate (PF) | 23 | 5 | Probable | Grade 3 | Definitely Preventable | Cured |
| P58 | 74 | F | Grade IV | Glaucoma | Latanoprost (BAC), Timolol (BAC), Diclofenac (BAC), Levofloxacin (BAC), PEG 400 | 24 | 5 | Probable | Grade 3 | Definitely Preventable | Cured |
| P59 | 59 | F | Grade IV | Dry Eye | Tobramycin (BAC), Dex (BAC), Diclofenac (BAC), Ganciclovir (BAC), Hyaluronate | 28 | 5 | Possible | Grade 3 | Probably Preventable | Cured |
| P60 | 72 | M | Grade IV | Glaucoma | Timolol (BAC), Brimonidine (Purite), Latanoprost (BAC), Levofloxacin (BAC), Dex (BAC) | 30 | 5 | Probable | Grade 3 | Definitely Preventable | Improved |

BAC: Benzalkonium chloride; Conc.: Concentration; Dex: Dexamethasone; MI-PCED: Medication-induced persistent corneal epithelial defects; n: Number of concurrent medications; PEG: Polyethylene glycol; PF: Preservative-free; WHO-UMC: World Health Organization-Uppsala Monitoring Centre; F:Female; M: Male
